# 3-Hexadecyl-1,5-dimethyl-1*H*-1,5-benzo­diazepine-2,4(3*H*,5*H*)-dione

**DOI:** 10.1107/S1600536811013663

**Published:** 2011-04-16

**Authors:** Rachida Dardouri, Youssef Kandri Rodi, Sonia Ladeira, El Mokhtar Essassi, Seik Weng Ng

**Affiliations:** a Laboratoire de Chimie Organique Hétérocyclique, Pôle de Compétences Pharmacochimie, Université Mohammed V-Agdal, BP 1014 Avenue Ibn Batout, Rabat, Morocco; b Service Commun Rayons-X, Laboratoire de Chimie de Coordination, Toulouse, France; c Department of Chemistry, University of Malaya, 50603 Kuala Lumpur, Malaysia

## Abstract

In the title mol­ecule, C_27_H_44_N_2_O_2_, the seven-membered ring adopts a boat-shaped conformation, with two C atoms of the fused benzene ring forming the stern and the methine C atom forming the prow. The hexa­decyl substituent occupies an equatorial position, with the aliphatic chain exhibibiting an extended zigzag conformation.

## Related literature

For the 3-tetra­decyl-substituted analog, see: Dardouri *et al.* (2011 ▶).
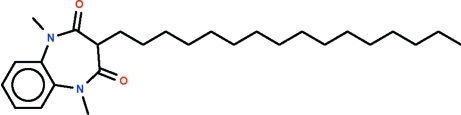


## Experimental

### 

#### Crystal data

C_27_H_44_N_2_O_2_
*M* *_r_* = 428.64
Monoclinic, 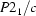

*a* = 8.1426 (1) Å
*b* = 36.2705 (5) Å
*c* = 9.4090 (1) Å
β = 114.611 (1)°
*V* = 2526.38 (5) Å^3^
*Z* = 4
Mo *K*α radiation
μ = 0.07 mm^−1^
*T* = 293 K
0.50 × 0.30 × 0.10 mm

#### Data collection

Bruker X8 APEXII diffractometer
45223 measured reflections
7353 independent reflections
5105 reflections with *I* > 2σ(*I*)
*R*_int_ = 0.036

#### Refinement

*R*[*F* ^2^ > 2σ(*F* ^2^)] = 0.050
*wR*(*F* ^2^) = 0.159
*S* = 1.03
7353 reflections
282 parameters
H-atom parameters constrained
Δρ_max_ = 0.34 e Å^−3^
Δρ_min_ = −0.26 e Å^−3^

### 

Data collection: *APEX2* (Bruker, 2008 ▶); cell refinement: *SAINT* (Bruker, 2008 ▶); data reduction: *SAINT*; program(s) used to solve structure: *SHELXS97* (Sheldrick, 2008 ▶); program(s) used to refine structure: *SHELXL97* (Sheldrick, 2008 ▶); molecular graphics: *X-SEED* (Barbour, 2001 ▶); software used to prepare material for publication: *publCIF* (Westrip, 2010 ▶).

## Supplementary Material

Crystal structure: contains datablocks global, I. DOI: 10.1107/S1600536811013663/lh5231sup1.cif

Structure factors: contains datablocks I. DOI: 10.1107/S1600536811013663/lh5231Isup2.hkl

Additional supplementary materials:  crystallographic information; 3D view; checkCIF report

## Figures and Tables

**Fig. 1.**
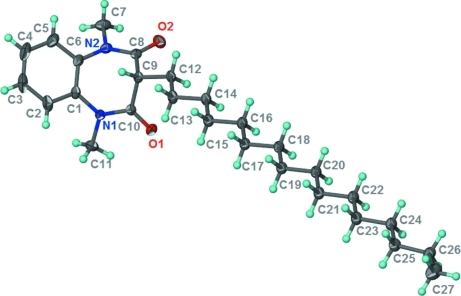
Thermal ellipsoid plot (Barbour, 2001) of C27H44N2O2 at the 50% probability level; hydrogen atoms are drawn as arbitrary radius.

## supplementary crystallographic information

### Comment

The methylene part of 1,5-dimethyl-1,5-benzodiazepine-2,4-dione is relatively acidic, and one proton can be abstracted by using potassium *t*-butoxide. The resulting carbanion can undergo a nucleophlilic subsitution with a dibromoalkane to form a 3-substituted derivative. In a previous study, the compound was reacted with 1-bromotetradecane to give the tetradecyl substitued derivative (Dardouri *et al.*, 2011). The corresponding hexdecyl title compound (Fig. 1) was obtained by using 1-bromohexadecane.

### Experimental

To a solution of the potassium *t*-butoxide (0.42 g, 3.6 mmol) in DMF (15 ml) was added 1,5-dimethyl-1,5-benzodiazepine-2,4-dione (0.50 g, 2.4 mmol) and 1-bromotetradecane (0.88 ml, 2.88 mmol). Stirring was continued for 24 h. The reaction was monitored by thin layer chromatography. The mixture was filtered and the solution evaporated to give colorless crystals.

### Refinement

Carbon-bound H-atoms were placed in calculated positions (C—H 0.93–0.98 Å) and were included in the refinement in the riding model approximation, with *U*(H) set to 1.2–1.5*U*_eq_(C). Two reflections with bad agreements (0 2 0, 0 4 0) were omitted from the refinemnt.

### Crystal data

**Table:**

C_27_H_44_N_2_O_2_	*F*(000) = 944
*M**_r_* = 428.64	*D*_x_ = 1.127 Mg m^−^^3^
Monoclinic, *P*2_1_/*c*	Mo *K*α radiation, λ = 0.71073 Å
Hall symbol: -P 2ybc	Cell parameters from 9990 reflections
*a* = 8.1426 (1) Å	θ = 2.6–30.0°
*b* = 36.2705 (5) Å	µ = 0.07 mm^−^^1^
*c* = 9.4090 (1) Å	*T* = 293 K
β = 114.611 (1)°	Plate, colorless
*V* = 2526.38 (5) Å^3^	0.50 × 0.30 × 0.10 mm
*Z* = 4	

### Data collection

**Table:**

Bruker X8 APEXII diffractometer	5105 reflections with *I* > 2σ(*I*)
Radiation source: fine-focus sealed tube	*R*_int_ = 0.036
graphite	θ_max_ = 30.0°, θ_min_ = 2.5°
φ and ω scans	*h* = −10→11
45223 measured reflections	*k* = −50→51
7353 independent reflections	*l* = −12→13

### Refinement

**Table:**

Refinement on *F*^2^	Primary atom site location: structure-invariant direct methods
Least-squares matrix: full	Secondary atom site location: difference Fourier map
*R*[*F*^2^ > 2σ(*F*^2^)] = 0.050	Hydrogen site location: inferred from neighbouring sites
*wR*(*F*^2^) = 0.159	H-atom parameters constrained
*S* = 1.03	*w* = 1/[σ^2^(*F*_o_^2^) + (0.0871*P*)^2^ + 0.3018*P*] where *P* = (*F*_o_^2^ + 2*F*_c_^2^)/3
7353 reflections	(Δ/σ)_max_ = 0.001
282 parameters	Δρ_max_ = 0.34 e Å^−^^3^
0 restraints	Δρ_min_ = −0.26 e Å^−^^3^

### Fractional atomic coordinates and isotropic or equivalent isotropic displacement parameters (Å^2^)

**Table:**

	*x*	*y*	*z*	*U*_iso_*/*U*_eq_	
O1	0.02825 (12)	0.20901 (2)	0.37201 (10)	0.0323 (2)	
O2	−0.39162 (13)	0.17141 (3)	0.40983 (10)	0.0376 (2)	
N1	−0.19184 (13)	0.22225 (3)	0.13447 (11)	0.0271 (2)	
N2	−0.51023 (13)	0.19449 (3)	0.16414 (12)	0.0268 (2)	
C1	−0.35060 (17)	0.21187 (3)	0.00186 (14)	0.0273 (3)	
C2	−0.3522 (2)	0.21567 (4)	−0.14654 (15)	0.0400 (3)	
H2	−0.2498	0.2243	−0.1560	0.048*	
C3	−0.5039 (2)	0.20671 (4)	−0.27923 (16)	0.0491 (4)	
H3	−0.5028	0.2088	−0.3773	0.059*	
C4	−0.6573 (2)	0.19462 (4)	−0.26542 (17)	0.0485 (4)	
H4	−0.7606	0.1891	−0.3546	0.058*	
C5	−0.65797 (19)	0.19072 (3)	−0.11957 (16)	0.0384 (3)	
H5	−0.7621	0.1825	−0.1117	0.046*	
C6	−0.50464 (16)	0.19891 (3)	0.01618 (14)	0.0267 (3)	
C7	−0.67424 (18)	0.20524 (4)	0.18255 (19)	0.0388 (3)	
H7A	−0.6415	0.2151	0.2855	0.058*	
H7B	−0.7501	0.1840	0.1681	0.058*	
H7C	−0.7383	0.2236	0.1061	0.058*	
C8	−0.37885 (16)	0.17653 (3)	0.28661 (13)	0.0249 (2)	
C9	−0.21270 (15)	0.16463 (3)	0.26138 (13)	0.0233 (2)	
H9A	−0.2522	0.1529	0.1587	0.028*	
C10	−0.11159 (16)	0.20013 (3)	0.26219 (13)	0.0237 (2)	
C11	−0.09947 (19)	0.25653 (4)	0.12803 (18)	0.0384 (3)	
H11A	−0.0195	0.2638	0.2321	0.058*	
H11B	−0.1873	0.2756	0.0806	0.058*	
H11C	−0.0312	0.2526	0.0672	0.058*	
C12	−0.09467 (16)	0.13780 (3)	0.38687 (13)	0.0268 (3)	
H12A	−0.1690	0.1176	0.3940	0.032*	
H12B	−0.0452	0.1504	0.4870	0.032*	
C13	0.06032 (17)	0.12220 (3)	0.35425 (14)	0.0304 (3)	
H13A	0.0108	0.1073	0.2601	0.036*	
H13B	0.1266	0.1424	0.3354	0.036*	
C14	0.19019 (17)	0.09876 (3)	0.48780 (14)	0.0279 (3)	
H14A	0.1243	0.0782	0.5046	0.034*	
H14B	0.2367	0.1134	0.5826	0.034*	
C15	0.34868 (17)	0.08387 (3)	0.45879 (14)	0.0295 (3)	
H15A	0.3025	0.0687	0.3655	0.035*	
H15B	0.4133	0.1044	0.4398	0.035*	
C16	0.47967 (16)	0.06121 (3)	0.59449 (14)	0.0283 (3)	
H16A	0.4146	0.0409	0.6139	0.034*	
H16B	0.5262	0.0765	0.6875	0.034*	
C17	0.63784 (16)	0.04591 (3)	0.56685 (14)	0.0278 (3)	
H17A	0.7022	0.0662	0.5462	0.033*	
H17B	0.5914	0.0304	0.4746	0.033*	
C18	0.77024 (16)	0.02355 (3)	0.70403 (14)	0.0278 (3)	
H18A	0.8161	0.0390	0.7964	0.033*	
H18B	0.7060	0.0031	0.7243	0.033*	
C19	0.92947 (16)	0.00835 (3)	0.67688 (14)	0.0277 (3)	
H19A	0.8839	−0.0079	0.5869	0.033*	
H19B	0.9910	0.0287	0.6527	0.033*	
C20	1.06521 (16)	−0.01274 (3)	0.81635 (14)	0.0292 (3)	
H20A	1.1111	0.0036	0.9062	0.035*	
H20B	1.0035	−0.0330	0.8408	0.035*	
C21	1.22377 (17)	−0.02808 (3)	0.78946 (14)	0.0297 (3)	
H21A	1.2842	−0.0079	0.7631	0.036*	
H21B	1.1782	−0.0448	0.7009	0.036*	
C22	1.36085 (17)	−0.04850 (4)	0.93023 (15)	0.0319 (3)	
H22A	1.3000	−0.0685	0.9571	0.038*	
H22B	1.4070	−0.0317	1.0185	0.038*	
C23	1.51950 (17)	−0.06420 (4)	0.90372 (14)	0.0317 (3)	
H23A	1.5760	−0.0445	0.8706	0.038*	
H23B	1.4744	−0.0822	0.8199	0.038*	
C24	1.66148 (17)	−0.08248 (3)	1.04886 (14)	0.0309 (3)	
H24A	1.7070	−0.0644	1.1323	0.037*	
H24B	1.6045	−0.1020	1.0824	0.037*	
C25	1.81995 (17)	−0.09856 (4)	1.02357 (15)	0.0318 (3)	
H25A	1.7764	−0.1184	0.9479	0.038*	
H25B	1.8696	−0.0796	0.9800	0.038*	
C26	1.96936 (17)	−0.11326 (3)	1.17273 (15)	0.0331 (3)	
H26A	2.0196	−0.0929	1.2450	0.040*	
H26B	1.9174	−0.1306	1.2210	0.040*	
C27	2.1211 (2)	−0.13226 (5)	1.1471 (2)	0.0527 (4)	
H27A	2.2105	−0.1408	1.2454	0.079*	
H27B	2.1755	−0.1151	1.1017	0.079*	
H27C	2.0733	−0.1528	1.0778	0.079*	

### Atomic displacement parameters (Å^2^)

**Table:**

	*U*^11^	*U*^22^	*U*^33^	*U*^12^	*U*^13^	*U*^23^
O1	0.0250 (5)	0.0383 (5)	0.0285 (5)	−0.0006 (4)	0.0060 (4)	0.0013 (4)
O2	0.0388 (6)	0.0456 (5)	0.0359 (5)	0.0037 (4)	0.0231 (4)	0.0053 (4)
N1	0.0210 (5)	0.0307 (5)	0.0285 (5)	0.0020 (4)	0.0092 (4)	0.0081 (4)
N2	0.0197 (5)	0.0258 (5)	0.0348 (5)	0.0031 (4)	0.0112 (4)	0.0009 (4)
C1	0.0275 (6)	0.0265 (6)	0.0246 (5)	0.0091 (5)	0.0074 (5)	0.0048 (4)
C2	0.0447 (8)	0.0452 (8)	0.0309 (7)	0.0174 (6)	0.0166 (6)	0.0137 (6)
C3	0.0656 (11)	0.0486 (9)	0.0249 (7)	0.0224 (8)	0.0106 (7)	0.0087 (6)
C4	0.0545 (10)	0.0342 (7)	0.0314 (7)	0.0090 (7)	−0.0074 (7)	−0.0003 (6)
C5	0.0319 (7)	0.0271 (6)	0.0408 (7)	0.0028 (5)	0.0000 (6)	0.0011 (5)
C6	0.0249 (6)	0.0203 (5)	0.0288 (6)	0.0065 (4)	0.0052 (5)	0.0019 (4)
C7	0.0238 (7)	0.0365 (7)	0.0601 (9)	0.0034 (5)	0.0215 (7)	0.0009 (6)
C8	0.0238 (6)	0.0231 (5)	0.0279 (6)	−0.0004 (4)	0.0110 (5)	−0.0012 (4)
C9	0.0223 (6)	0.0246 (5)	0.0216 (5)	0.0048 (4)	0.0080 (4)	0.0023 (4)
C10	0.0220 (6)	0.0276 (5)	0.0236 (5)	0.0057 (4)	0.0116 (5)	0.0027 (4)
C11	0.0294 (7)	0.0373 (7)	0.0492 (8)	−0.0004 (5)	0.0171 (6)	0.0144 (6)
C12	0.0269 (6)	0.0282 (6)	0.0248 (5)	0.0070 (5)	0.0102 (5)	0.0076 (4)
C13	0.0315 (7)	0.0328 (6)	0.0271 (6)	0.0122 (5)	0.0125 (5)	0.0085 (5)
C14	0.0280 (6)	0.0282 (6)	0.0273 (6)	0.0082 (5)	0.0111 (5)	0.0059 (5)
C15	0.0283 (7)	0.0311 (6)	0.0292 (6)	0.0088 (5)	0.0120 (5)	0.0067 (5)
C16	0.0247 (6)	0.0318 (6)	0.0274 (6)	0.0073 (5)	0.0098 (5)	0.0049 (5)
C17	0.0244 (6)	0.0303 (6)	0.0275 (6)	0.0067 (5)	0.0097 (5)	0.0042 (5)
C18	0.0248 (6)	0.0297 (6)	0.0289 (6)	0.0060 (5)	0.0111 (5)	0.0054 (5)
C19	0.0247 (6)	0.0295 (6)	0.0283 (6)	0.0070 (5)	0.0103 (5)	0.0037 (5)
C20	0.0254 (6)	0.0327 (6)	0.0294 (6)	0.0069 (5)	0.0113 (5)	0.0067 (5)
C21	0.0269 (6)	0.0341 (6)	0.0280 (6)	0.0081 (5)	0.0113 (5)	0.0055 (5)
C22	0.0274 (7)	0.0370 (7)	0.0319 (6)	0.0096 (5)	0.0128 (5)	0.0077 (5)
C23	0.0284 (7)	0.0367 (6)	0.0303 (6)	0.0103 (5)	0.0124 (5)	0.0062 (5)
C24	0.0281 (7)	0.0342 (6)	0.0306 (6)	0.0084 (5)	0.0124 (5)	0.0048 (5)
C25	0.0291 (7)	0.0332 (6)	0.0341 (6)	0.0090 (5)	0.0140 (6)	0.0044 (5)
C26	0.0275 (7)	0.0293 (6)	0.0374 (7)	0.0039 (5)	0.0083 (6)	0.0017 (5)
C27	0.0346 (8)	0.0525 (9)	0.0647 (10)	0.0169 (7)	0.0145 (8)	0.0033 (8)

### Geometric parameters (Å, °)

**Table:**

O1—C10	1.2197 (14)	C15—H15A	0.9700
O2—C8	1.2206 (14)	C15—H15B	0.9700
N1—C10	1.3640 (14)	C16—C17	1.5209 (16)
N1—C1	1.4235 (16)	C16—H16A	0.9700
N1—C11	1.4676 (16)	C16—H16B	0.9700
N2—C8	1.3676 (15)	C17—C18	1.5254 (16)
N2—C6	1.4208 (15)	C17—H17A	0.9700
N2—C7	1.4690 (16)	C17—H17B	0.9700
C1—C6	1.3975 (18)	C18—C19	1.5252 (16)
C1—C2	1.3976 (17)	C18—H18A	0.9700
C2—C3	1.380 (2)	C18—H18B	0.9700
C2—H2	0.9300	C19—C20	1.5233 (16)
C3—C4	1.380 (2)	C19—H19A	0.9700
C3—H3	0.9300	C19—H19B	0.9700
C4—C5	1.382 (2)	C20—C21	1.5207 (16)
C4—H4	0.9300	C20—H20A	0.9700
C5—C6	1.3964 (18)	C20—H20B	0.9700
C5—H5	0.9300	C21—C22	1.5223 (17)
C7—H7A	0.9600	C21—H21A	0.9700
C7—H7B	0.9600	C21—H21B	0.9700
C7—H7C	0.9600	C22—C23	1.5244 (17)
C8—C9	1.5298 (16)	C22—H22A	0.9700
C9—C12	1.5230 (15)	C22—H22B	0.9700
C9—C10	1.5266 (16)	C23—C24	1.5251 (17)
C9—H9A	0.9800	C23—H23A	0.9700
C11—H11A	0.9600	C23—H23B	0.9700
C11—H11B	0.9600	C24—C25	1.5222 (17)
C11—H11C	0.9600	C24—H24A	0.9700
C12—C13	1.5264 (16)	C24—H24B	0.9700
C12—H12A	0.9700	C25—C26	1.5205 (17)
C12—H12B	0.9700	C25—H25A	0.9700
C13—C14	1.5210 (16)	C25—H25B	0.9700
C13—H13A	0.9700	C26—C27	1.5196 (19)
C13—H13B	0.9700	C26—H26A	0.9700
C14—C15	1.5248 (16)	C26—H26B	0.9700
C14—H14A	0.9700	C27—H27A	0.9600
C14—H14B	0.9700	C27—H27B	0.9600
C15—C16	1.5195 (16)	C27—H27C	0.9600
			
C10—N1—C1	122.93 (10)	C15—C16—C17	113.69 (10)
C10—N1—C11	117.97 (10)	C15—C16—H16A	108.8
C1—N1—C11	118.81 (10)	C17—C16—H16A	108.8
C8—N2—C6	123.12 (10)	C15—C16—H16B	108.8
C8—N2—C7	117.24 (10)	C17—C16—H16B	108.8
C6—N2—C7	119.16 (10)	H16A—C16—H16B	107.7
C6—C1—C2	119.60 (12)	C16—C17—C18	113.56 (10)
C6—C1—N1	121.90 (10)	C16—C17—H17A	108.9
C2—C1—N1	118.48 (12)	C18—C17—H17A	108.9
C3—C2—C1	120.87 (15)	C16—C17—H17B	108.9
C3—C2—H2	119.6	C18—C17—H17B	108.9
C1—C2—H2	119.6	H17A—C17—H17B	107.7
C4—C3—C2	119.58 (14)	C19—C18—C17	113.64 (10)
C4—C3—H3	120.2	C19—C18—H18A	108.8
C2—C3—H3	120.2	C17—C18—H18A	108.8
C3—C4—C5	120.28 (14)	C19—C18—H18B	108.8
C3—C4—H4	119.9	C17—C18—H18B	108.8
C5—C4—H4	119.9	H18A—C18—H18B	107.7
C4—C5—C6	120.98 (14)	C20—C19—C18	113.56 (10)
C4—C5—H5	119.5	C20—C19—H19A	108.9
C6—C5—H5	119.5	C18—C19—H19A	108.9
C5—C6—C1	118.67 (12)	C20—C19—H19B	108.9
C5—C6—N2	119.41 (12)	C18—C19—H19B	108.9
C1—C6—N2	121.91 (11)	H19A—C19—H19B	107.7
N2—C7—H7A	109.5	C21—C20—C19	113.71 (10)
N2—C7—H7B	109.5	C21—C20—H20A	108.8
H7A—C7—H7B	109.5	C19—C20—H20A	108.8
N2—C7—H7C	109.5	C21—C20—H20B	108.8
H7A—C7—H7C	109.5	C19—C20—H20B	108.8
H7B—C7—H7C	109.5	H20A—C20—H20B	107.7
O2—C8—N2	121.78 (11)	C20—C21—C22	113.51 (10)
O2—C8—C9	122.36 (11)	C20—C21—H21A	108.9
N2—C8—C9	115.83 (10)	C22—C21—H21A	108.9
C12—C9—C10	111.73 (10)	C20—C21—H21B	108.9
C12—C9—C8	111.91 (9)	C22—C21—H21B	108.9
C10—C9—C8	105.74 (9)	H21A—C21—H21B	107.7
C12—C9—H9A	109.1	C21—C22—C23	113.81 (10)
C10—C9—H9A	109.1	C21—C22—H22A	108.8
C8—C9—H9A	109.1	C23—C22—H22A	108.8
O1—C10—N1	121.80 (11)	C21—C22—H22B	108.8
O1—C10—C9	122.55 (10)	C23—C22—H22B	108.8
N1—C10—C9	115.60 (10)	H22A—C22—H22B	107.7
N1—C11—H11A	109.5	C22—C23—C24	113.20 (10)
N1—C11—H11B	109.5	C22—C23—H23A	108.9
H11A—C11—H11B	109.5	C24—C23—H23A	108.9
N1—C11—H11C	109.5	C22—C23—H23B	108.9
H11A—C11—H11C	109.5	C24—C23—H23B	108.9
H11B—C11—H11C	109.5	H23A—C23—H23B	107.8
C9—C12—C13	112.87 (9)	C25—C24—C23	113.73 (10)
C9—C12—H12A	109.0	C25—C24—H24A	108.8
C13—C12—H12A	109.0	C23—C24—H24A	108.8
C9—C12—H12B	109.0	C25—C24—H24B	108.8
C13—C12—H12B	109.0	C23—C24—H24B	108.8
H12A—C12—H12B	107.8	H24A—C24—H24B	107.7
C14—C13—C12	112.94 (9)	C26—C25—C24	113.24 (10)
C14—C13—H13A	109.0	C26—C25—H25A	108.9
C12—C13—H13A	109.0	C24—C25—H25A	108.9
C14—C13—H13B	109.0	C26—C25—H25B	108.9
C12—C13—H13B	109.0	C24—C25—H25B	108.9
H13A—C13—H13B	107.8	H25A—C25—H25B	107.7
C13—C14—C15	113.52 (10)	C27—C26—C25	113.80 (12)
C13—C14—H14A	108.9	C27—C26—H26A	108.8
C15—C14—H14A	108.9	C25—C26—H26A	108.8
C13—C14—H14B	108.9	C27—C26—H26B	108.8
C15—C14—H14B	108.9	C25—C26—H26B	108.8
H14A—C14—H14B	107.7	H26A—C26—H26B	107.7
C16—C15—C14	113.04 (10)	C26—C27—H27A	109.5
C16—C15—H15A	109.0	C26—C27—H27B	109.5
C14—C15—H15A	109.0	H27A—C27—H27B	109.5
C16—C15—H15B	109.0	C26—C27—H27C	109.5
C14—C15—H15B	109.0	H27A—C27—H27C	109.5
H15A—C15—H15B	107.8	H27B—C27—H27C	109.5
			
C10—N1—C1—C6	50.83 (16)	N2—C8—C9—C10	70.80 (12)
C11—N1—C1—C6	−135.46 (12)	C1—N1—C10—O1	177.18 (11)
C10—N1—C1—C2	−130.67 (12)	C11—N1—C10—O1	3.42 (17)
C11—N1—C1—C2	43.04 (16)	C1—N1—C10—C9	−5.49 (16)
C6—C1—C2—C3	−0.15 (19)	C11—N1—C10—C9	−179.24 (10)
N1—C1—C2—C3	−178.69 (12)	C12—C9—C10—O1	−16.10 (15)
C1—C2—C3—C4	1.4 (2)	C8—C9—C10—O1	105.89 (12)
C2—C3—C4—C5	−1.4 (2)	C12—C9—C10—N1	166.59 (9)
C3—C4—C5—C6	0.0 (2)	C8—C9—C10—N1	−71.43 (12)
C4—C5—C6—C1	1.23 (18)	C10—C9—C12—C13	−68.09 (13)
C4—C5—C6—N2	−179.85 (11)	C8—C9—C12—C13	173.55 (10)
C2—C1—C6—C5	−1.16 (17)	C9—C12—C13—C14	173.66 (10)
N1—C1—C6—C5	177.32 (11)	C12—C13—C14—C15	−178.37 (10)
C2—C1—C6—N2	179.94 (11)	C13—C14—C15—C16	178.71 (11)
N1—C1—C6—N2	−1.57 (17)	C14—C15—C16—C17	179.55 (10)
C8—N2—C6—C5	132.06 (12)	C15—C16—C17—C18	179.34 (10)
C7—N2—C6—C5	−39.70 (15)	C16—C17—C18—C19	−179.70 (10)
C8—N2—C6—C1	−49.05 (16)	C17—C18—C19—C20	177.81 (10)
C7—N2—C6—C1	139.19 (12)	C18—C19—C20—C21	179.76 (10)
C6—N2—C8—O2	−176.04 (11)	C19—C20—C21—C22	178.91 (11)
C7—N2—C8—O2	−4.13 (17)	C20—C21—C22—C23	179.57 (11)
C6—N2—C8—C9	6.17 (15)	C21—C22—C23—C24	176.41 (11)
C7—N2—C8—C9	178.07 (10)	C22—C23—C24—C25	179.53 (11)
O2—C8—C9—C12	14.88 (16)	C23—C24—C25—C26	174.22 (11)
N2—C8—C9—C12	−167.34 (10)	C24—C25—C26—C27	175.17 (12)
O2—C8—C9—C10	−106.98 (12)		

## References

[bb1] Barbour, L. J. (2001). *J. Supramol. Chem.* **1**, 189–191.

[bb2] Bruker (2008). *APEX2* and *SAINT* Bruker AXS Inc., Madison, Wisconsin, USA.

[bb3] Dardouri, R., Ouazzani Chahdi, F., Saffon, N., Essassi, E. M. & Ng, S. W. (2011). *Acta Cryst.* E**67**, o674. 10.1107/S1600536811005782 PMC3052003 21522422

[bb4] Sheldrick, G. M. (2008). *Acta Cryst.* A**64**, 112–122. 10.1107/S0108767307043930 18156677

[bb5] Westrip, S. P. (2010). *J. Appl. Cryst.* **43**, 920–925.

